# Relationship between HbA1c, fructosamine and clinical assessment of glycemic control in dogs

**DOI:** 10.1371/journal.pone.0264275

**Published:** 2022-02-25

**Authors:** Olga Norris, Thomas Schermerhorn

**Affiliations:** Department of Clinical Sciences, College of Veterinary Medicine, Kansas State University, Manhattan, KS, United States of America; University of Lincoln, UNITED KINGDOM

## Abstract

**Background:**

Serum fructosamine is a routine test used for clinical monitoring of diabetes mellitus (DM) but the usefulness of HbA1c for this purpose has not been extensively studied.

**Hypothesis:**

The study aimed to compare the ability of blood HbA1c and serum fructosamine tests to correctly classify DM control determined using a clinically-based assessment.

**Animals:**

28 client-owned dogs with naturally-occurring diabetes mellitus.

**Methods:**

Cross-sectional observational study. Ability of fructosamine and HbA1c tests to classify diabetes control in dogs was determined.

**Results:**

Clinical assessment classified 50% of dogs as having good diabetic control and 82% as having acceptable diabetic control. Analysis using Cohen’s kappa test showed that agreements between fructosamine and HbA1c results and the clinical assessment ranged from poor to fair. Fructosamine and HbA1c results from each dog showed a moderate correlation. Overall, the HbA1c test showed the best agreement with the clinical assessment when diabetes control was considered either acceptable or unacceptable, although the strength of agreement was considered fair (kappa = 0.27).

**Conclusions and clinical importance:**

The HbA1c concentration was found to be more consistent with clinical evaluation of diabetes control than was the serum fructosamine concentration. The HbA1c level is a useful tool for assessment of glycemic status in diabetic dogs but should be used alongside other tests for outpatient monitoring of clinically stable diabetic dogs.

## Introduction

Effective monitoring of diabetes mellitus (DM) aims to assess glycemic control using combined clinical and laboratory evaluation, including measurement of blood glycated proteins. Published guidelines from the American Animal Hospital Association emphasizes the need for periodic monitoring to ensure control of clinical signs, which is a major goal of clinical management of DM [1]. Minimizing or eliminating clinical signs improves patient quality of life and pet-owner satisfaction and has provided a standard for clinical studies to evaluate diabetes treatment and control [2, 3].

In the presence of hyperglycemia, serum proteins undergo an irreversible, permanent, non-enzymatic chemical glycation reaction that produces a glycated protein moiety which maintains functional characteristics of the parent protein [4]. The circulating concentration of a specific glycated protein depends on the half-life of the parent protein and correlates with the prevailing blood glucose concentration [4].

Serum fructosamine is frequently evaluated to assess diabetic control in dogs [1]. The serum fructosamine test measures a pool of glycated proteins, including glycated albumin, which comprises the bulk of the measured fructosamine concentration [4]. In the dog, the fructosamine concentration is interpreted to represent the average blood glucose concentration over the two weeks prior to testing, a time frame that approximates the lifespan of serum albumin [1].

Blood hemoglobin A1c (HbA1c), a specific glycated hemoglobin moiety, is extensively used as a diabetes control marker in people but is less studied in dogs [5, 6]. The HbA1c moiety measured in assays is produced by stable glycation of the amino terminal valine residue of the beta chain of hemoglobin A, which comprises 97% of circulating hemoglobin in humans [7]. The extended life span of hemoglobin in circulation compared with albumin means that the HbA1c concentration reflects the blood glucose concentration over a substantially longer time frame than the fructosamine concentration. Accordingly, HbA1c reflects the average glucose concentration for the previous 120 days in dogs, the approximate lifespan of canine hemoglobin [8]. Recent studies have concluded that blood HbA1c may have a useful role in glycemic assessment in diabetic dogs [6, 8–10]. Despite encouraging results, limited availability of HbA1c testing for dogs has hampered its routine use in diabetes monitoring protocols. Recently, a commercial canine HbA1c assay (A1Care, Baycom Diagnostics) has made HbA1c testing more accessible for routine clinical use.

The study aimed to compare the ability of blood HbA1c and serum fructosamine tests to correctly classify DM control determined using a clinically-based assessment.

## Materials and methods

Diabetic dogs from an outpatient population examined at a university teaching hospital were consecutively enrolled through a 1-year period beginning October, 2018. Inclusion requirements were: 1) a previous diagnosis of diabetes mellitus; 2) insulin treatment had been given for a least 1-month; 3) the dog was clinically stable. For purposes of the study, a dog was considered clinically stable if it was being maintained as an outpatient, the owner had no concerns about the dog’s current health status (other than diabetes mellitus) and any pre-existing chronic disorders were satisfactorily controlled. Newly diagnosed diabetic dogs and diabetic dogs with evidence of complicated diabetes (diabetic ketoacidosis or hyperglycemic hyperosmolar syndrome) or concurrent illness that warranted hospitalization were excluded. The targeted enrollment was determined by calculating the number of dogs needed to determine separate population means for fructosamine and HbA1c. For HbA1c, calculation of mean with 95% CI using an estimated standard deviation of the value in the population of +/- 5 and a specified precision of the estimate of 2 yielded a minimum group size of 25 dogs. For fructosamine calculation of mean with 95% CI using an estimated standard deviation of the value in the population of +/- 200 and a specified precision of the estimate of 100 yielded a minimum group size of 25 dogs. The study was performed in accordance with the Kansas State University, College of Veterinary Medicine guidelines for animal research and was reviewed by the Institutional Animal Care and Use Committee.

A laboratory evaluation including a serum glucose and albumin concentrations, serum fructosamine concentration, and blood HbA1c concentration was completed for each dog. In addition, the packed red blood cell volume (PCV) and total protein was measured for each dog. Serum glucose and albumin were measured using a COBAS C501 Chemistry Analyzer (Roche Diagnostics; Indianapolis, IN) by the Clinical Pathology Laboratory of the Kansas State University, College of Veterinary Medicine. Serum glucose was performed as part of routine evaluation of treated diabetic dogs for subclinical hypoglycemia. The PCV was obtained from a spun sample of whole blood that had been placed into a heparinized capillary tub. Serum total protein (TP) was determined using a refractometer. Serum fructosamine concentration (Antech Diagnostics) and HbA1c (A1Care, Baycom Diagnostics) were measured using commercial assays. Samples for serum fructosamine determination were prepared as follows: whole blood without added anticoagulant was allowed to clot, the clot was removed by centrifugation and the clarified serum removed by pipetting into a plastic tube and sent to the laboratory for assay. The serum fructosamine concentration was used to classify each dog’s diabetic control according to the following cut-off points provided by the laboratory: <360 nmol/L GOOD control, 360–442 nmol/L FAIR control, ≥443 nmol/L POOR control.

The A1CARE Assay (Baycom Diagnostics) is a commercial immunoassay for canine glycated hemoglobin (HbA1c). The assay has an average inter-assay CV of 9.6% and the average intra-Assay CV of 2.6%. During testing, species-specific Baycom manufactured controls are run on each assay plate for 2% glycation, 6% glycation, 8% glycation, 12% glycation and 30% glycation. Additional validation procedures use the Lyphochek Hemoglobin A1C Linearity Set Human A1C controls (Bio-Rad USA). Baycom Diagnostic’s A1CARE Assay uses a dried blood spot mail-in test manufactured at an FDA-approved facility and designed for use on canine and feline glycosylated hemoglobin. The Dried Blood Spot (DBS) methodology employs a DBS membrane supplied by Ahlstrom-Munksjo Grade 226 for sample collection and shipping. Samples for blood HbA1c determination were prepared according to instructions provided by the laboratory. Briefly, several drops of EDTA-anticoagulated whole blood were applied by pipette to each of four circular areas printed onto an absorbent DBS pad attached to the submission form. The absorbed blood was allowed to dry overnight at room temperature and the samples mailed to the laboratory. To perform the assay, a 3mm diameter disc is punched out into a 96 well plate using a DBS hole punch and 200 ul of phosphate-buffered saline containing 0.05% Tween 20 and 0.08% sodium azide added to each well containing a DBS sample. The sample plate is placed on a laboratory shaker at 1000 rpm for 30 minutes at room temperature to elute the sample from the disc. After elution, 6 ul of each sample elution is transferred to 4 separate wells of a 384-well plate to assure sample to sample intra-assay CV results are below 6% for each sample. As quickly as possible, 32 ul of reagent A (at 4 C) is added to each eluted sample and the plate is placed in an automated AIA-360 Automated Immunoassay Analyzer (TOSOH) where the sample is mixed (3 seconds) and incubated for 5 min at 37 C. After incubation, the plate is removed and 14 ul reagent B added to each well followed by another cycle of mixing (3 seconds) and incubation (5 min) at 37 C. After incubation is complete the plate is read at 720 nm. (G. Ray, personal communication). The laboratory presents the results of the HbA1c level as inconsistent or consistent with diabetes. HbA1c values from 0–4% are normal results, values from 4–6% represent ‘transitional’ results, and values >6% are consistent with diabetes. Since there is no information from dogs regarding expected HbA1c values associated with levels of diabetic control, we derived cut-off values for HbA1c using estimated average glucose (eAG) values. The eAG value for a given HbA1c value is derived from the mathematical relationship between glucose and HbA1c concentrations and was developed as a tool to help interpret HbA1c results [11]. Using the expected HbA1c value for non-diabetic dogs (<4%) as a starting point, the following formula was used to estimate HbA1c from a series of clinically relevant eAG concentrations: HbA1c = [eAG + 83]/43 (G. Ray, personal communication). The estimated average glucose (eAG) was calculated after rearranging the equation: eAG = [43 x HbA1c(%)]– 83. Cut-offs for blood HbA1c levels were determined: <4%-Good (eAG < 89 mg/dl), 4–8.5%—Fair (eAG 89–280 mg/dl), >8.5% Poor (eAG >280 mg/dl).

Clinical assessment consisted of physical examination and a Clinical Survey Tool (CST) that was completed during an interview with the dog owner. The CST consisted of 12 items with binary responses and concerned the 4-week period that preceded the interview [S1 File]. The CST was similar to a CST used previously to evaluate diabetic control in dogs [12]. Nine items required direct answers provided by dog owners to questions posed by the veterinarian completing the CST. These items recorded owners’ responses to questions about presence/absence of clinical signs, complications, and of treatment compliance. The remaining three items were independent of owner input and were recorded by the veterinarian supervising the evaluation and assessed specific parameters from the physical examination. Each item was assigned equal weight and scored either 0 or 1 for a maximal score of 12. The CST score classified diabetic control according to the scale: 11–12 = GOOD control, 7–10 = FAIR control, 0–6 = POOR control. Because the CST was dependent on the ability of the pet owner to recall specific events from memory, the cut-off points were selected to bias the CST score against returning a result of ‘good control’. Agreements between CST results and the result of each test (fructosamine and HbA1c) were examined using the Cohen kappa statistic. Two separate analyses were carried out. First, agreements between the CST result (GOOD, FAIR, or POOR) and results of each test (GOOD, FAIR, or POOR) were determined. In the second analysis, results of the CST and each test were re-classified as either ACCEPTABLE (combined GOOD and FAIR classifications) or UNACCEPTABLE (POOR classification) and agreements determined using binary classifications.

The CST score and laboratory test results (serum glucose, serum albumin, serum fructosamine, blood HbA1c, PCV and TP) were summarized and represented as median and range. Correlation between serum fructosamine concentrations and HbA1c concentrations was explored using Pearson’s R Test for Correlation and calculated using an online tool (https://www.socscistatistics.com/tests/pearson/default2.aspx) Agreement between the CST and test results were investigated using the Cohen kappa statistic and calculated using an online tool (https://www.graphpad.com/quickcalcs/kappa1) Agreements are presented as the kappa value with the estimated 95% confidence interval (CI).

## Results

Thirty dogs were enrolled but data sets for two dogs were incomplete: one dog was missing serum fructosamine data and one dog had an incomplete CST. Data from both dogs were excluded and analysis was carried out using complete data sets for the remaining dogs (n = 28). Signalment (age, breed), CST score, and laboratory results (serum glucose, serum fructosamine, and blood HbA1c) for individual dogs are provided as supporting information [S1 Dataset]. As a group, age (median 8.5 yr; range 6–14 yr) and breed varied among the study dogs. Insulin dose changes were allowed in the 1month preceding enrollment and were carried out under the supervision of the veterinary teams that cared for each patient. Fifteen dogs did not have any change in insulin dosing in the 1-month period prior to study enrollment. The shortest duration from the start of insulin therapy to study enrollment was 8 weeks. Summary statistics for serum fructosamine, blood HbA1c, and CST are shown in Table 1. Median serum glucose was 234 mg/dl (range 58–720 mg/dl). Serum glucose concentration exceeded the reference range in 21 dogs (75%) and hyperglycemia ranged from 127–720 mg/dl. Serum glucose was ≥ 200 mg/dl in seventeen dogs (57%). Five dogs had euglycemia and two dogs had mild hypoglycemia, which was subclinical. Median serum albumin was 3.6 g/dL (range 2.6–4.5 g/dL). Albumin was below the reference interval (3.2–4.2 g/dL) for 7 dogs and above the reference interval for 2 dogs. Median serum TP was 6.4 g/dL (range 4.6–6.4 g/dL). No dog had TP below the reference interval (5.3–6.9 g/dL) and 6 dogs had an increase in TP above the reference interval. Median PCV was 46% (range 30–54%). Six dogs had a PCV below the reference interval (41–59%); values for PCV in these 6 dogs ranged from 30–37%.

**Table 1:** Summary statistics for relevant clinical parameters.

	Median (Range)	Reference Range
Fructosamine (μmol/L)	425.5 (212–1173)	136–350*
Hb A1c (%)	5.45 (3.8–15.4)	≤ 4†
Clinical Score Tool	10.5 (5–12)	N/A

*The reference range for normal (non-diabetic) dogs is shown. The fructosamine concentration is expected to be elevated in most diabetic dogs. There is no established reference range for insulin-treated diabetic dogs but different cut-off values define different levels of glycemic control. Details about the cut-off values are found in the text.

†This value is the expected upper limit for %HbA1c in dogs that have a low likelihood of having DM. The HbA1c level is expected to be elevated in most diabetic dogs but cut-off and ranges for HbA1c that define different levels of glycemic control are not available. Details and rationale for the cut-off values used in the current study are found in the text.

N/A—not applicable.

The median CST was 10.5 (range 5–12). Dogs were classified as having either GOOD (n = 14; 50%), FAIR (n = 9; 32%), or POOR (n = 5; 18%) glucose control on the basis of the results of the CST. Classification of glycemic control by test result is shown in Table 2. The median serum fructosamine concentration was 425.5 nmol/ml (range 212–1173 nmol/ml). Dogs were classified on the basis of the serum fructosamine concentration as having GOOD (n = 7), FAIR (n = 8), or POOR (n = 13) glucose control. The median blood HbA1c concentration was 5.45% (3.8–15.4%). Dogs were classified on the basis of the blood HbA1c concentration as having GOOD (n = 4), FAIR (n = 19), or POOR (n = 5) glucose control.

**Table 2:** Classification of glycemic control by method.

	Method used for classification		
	CST	Fructosamine	HbA1c
Classification			
GOOD	14*	7	4
FAIR	9	8	19
POOR	5	13	5

*number in each cell represents *n* dogs with that result.

CST–Clinical Score Tool.

Glycated protein (fructosamine and hemoglobin A1c) measurements showed moderate correlation (Pearson’s R = 0.58; p = 0.001) Agreements between the CST result and results of fructosamine, HbA1c, or combined tests was poor (Table 3). Overall, the classification determined by CST was in agreement with the fructosamine result (but not the HbA1c result) for 7 dogs (25%; kappa -0.058, 95% CI -0.31–0.195) and with the HbA1c result (but not the fructosamine result) for 7 dogs (25%; kappa -0.105, 95% CI -0.358–0.148). Results of both tests agreed with the CST classification for only 2 dogs (5.5% kappa not performed). Disagreements were examined to identify dogs with test results that were in complete discordance with the CST. Ten dogs (35%) showed complete discordance between fructosamine and CST. In this group, the CST classification was GOOD and the fructosamine classification was POOR for 8 dogs while the CST classification was POOR and the fructosamine classification was GOOD for 2 dogs. The same analysis performed between CST and the HbA1c result identified just one dog (3.5%) with complete discordance (the CST classification was GOOD and the HbA1c classification was POOR). There were no dogs for which the CST showed complete discordance with results for both glycated protein tests.

**Table 3:** Agreement between glycated protein test results and CST classifications.

	Test			
	Fructosamine	HbA1c	Both	Neither
CST Classification				
GOOD	2*	1	1	10
FAIR	2	3	-	4
POOR	1	1	1	2

*number in each cell represents *n* dogs with a CST result (GOOD, FAIR, or POOR) that agrees with the result (GOOD, FAIR, or POOR) of individual or combined glycated protein tests.

“Both” indicates that each test yielded the same result and both were in agreement with the CST result.

“Neither” means that neither the fructosamine nor the HbA1c test result agreed with the CST result.

CST–Clinical Score Tool.

**Fig 1:**
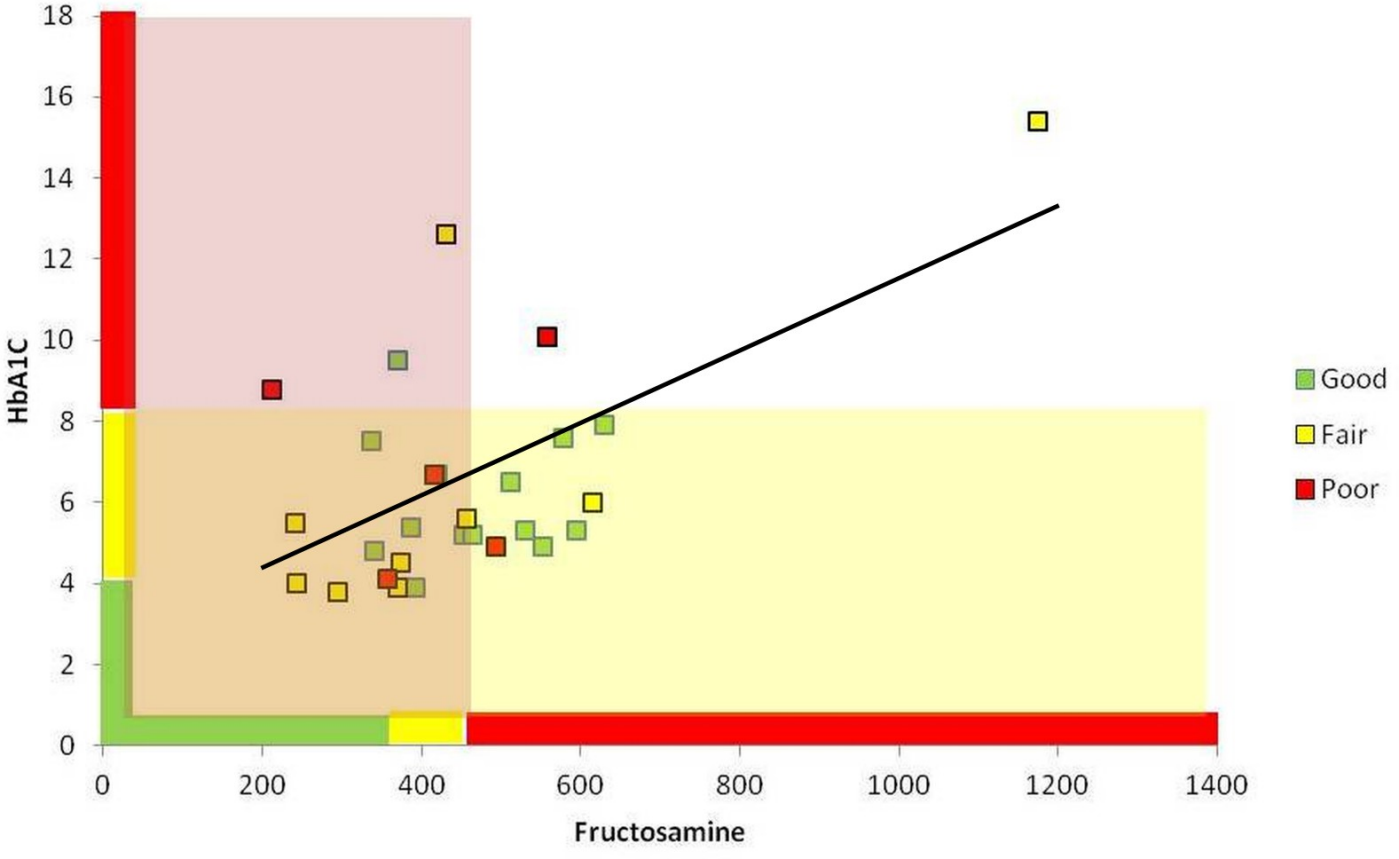
Relationships between classifications of glycemic control in study dogs by CST, blood HbA1c, and serum fructosamine. Individual dogs are represented by colored squares. The color of each square indicates glycemic classification determined using the gold standard (CST) assessment: GOOD–green, FAIR–yellow, POOR–red. The position of each square on the graph is determined by the dog’s serum fructosamine concentration (x-axis) and blood HbA1c concentration (y-axis). Respective cut-offs and ranges for classification using fructosamine and HbA1c are indicated by the color-coded bars along each axis (GOOD–green, FAIR–yellow, POOR–red). The pale red area indicates dogs classified as having ACCEPTABLE control by the serum fructosamine test. The pale-yellow area indicates dogs classified as having ACCEPTABLE control by the blood HbA1c test. Dogs that fall into the area of overlap (pale orange) were classified as having ACCEPTABLE control by both tests. Blood HbA1c (%) and serum fructosamine (μmol/l) concentrations showed a significant correlation among the study group (n = 28 dogs). Pearson’s R = 0.578; p = 0.001.

When CST and each test were classified as either ACCEPTABLE (combined GOOD and FAIR classifications) or UNACCEPTABLE (POOR classification), diabetic control was found to be acceptable for a majority (82%) of dogs (Fig 1). Overall, the CST classification (ACCEPTABLE or UNACCEPTABLE) was in agreement with the fructosamine result (ACCEPTABLE or UNACCEPTABLE) for 14 dogs (50%; kappa -0.048, 95% CI -0.342–0.246) and with the HbA1c result (ACCEPTABLE or UNACCEPTABLE) for 22 dogs (79%; kappa 0.270, 95% CI -0.170–0.709).

## Discussion

The results indicate that agreements between CST classification and glycated protein tests ranged from poor to fair. When the CST and test outcome had three possible classifications (GOOD, FAIR, or POOR), the CST showed no agreement with fructosamine or HbA1c results. When the CST and test outcome had two possible classifications (ACCEPTABLE or UNACCEPTABLE), agreement between the CST and HbA1c was considered ‘fair’ (kappa 0.27) while the CST and fructosamine showed no agreement (kappa -0.048). However, the associated confidence intervals for kappa for each test were wide, indicating these estimates are imprecise. Wide confidence intervals can result because the sampled population was small or because the measured target is variable within the population. The findings of the current study are consistent with those reported by Baldo et al. who employed a similar design but used different methods for data analysis to evaluate the clinical use of HbA1c and fructosamine in client-owned dogs with natural diabetes [6]. Using a clinical score-based assessment as the gold standard, they reported HbA1c and fructosamine tests had low sensitivities (41% and 61%, respectively) and moderate specificities (79% and 71%, respectively) for differentiating dogs with good glucose control from those with moderate or poor control [6].

Patient characteristics (age, sex, and breed) and routine laboratory test results (CBC and serum chemistry analysis) for the enrolled group were those expected for an outpatient population of insulin-treated dogs with chronic diabetes and consistent with those previously reported by studies that evaluated similar populations [13–16]. The observed high proportion of dogs with acceptable diabetic control was not surprising considering enrollment criteria specifically excluded dogs recently diagnosed with DM or those with complicated DM or other uncontrolled concurrent disorders. A wide range of blood HbA1c and fructosamine concentrations were recorded in the study dogs. Previous studies that employed different HbA1c and fructosamine assays and evaluated these proteins with respect to different levels of glycemic control reported values within a similar range, suggesting the assays used in the current study are providing useful clinical data [6, 8–10, 17].

It must be noted that temporal patterns of glycemia are reflected differently in changes in fructosamine and HbA1c concentrations and these differences likely affect interpretation of the study results. In completing the CST, owners were asked to provide information about the previous 4 weeks, a time frame that is longer than the period reflected by the fructosamine concentration and shorter than the period reflected by the HbA1c concentration. In selecting a time frame for evaluation, the 4-week time frame was chosen as a reasonable clinical window. However, short term changes (< 2 weeks) in glucose control might be under reported by some owners focused on describing signs over a longer reporting window. On the other hand, recent or worsening hyperglycemia that was accompanied by onset of noticeable clinical signs might weigh some owners’ recollections towards more proximal changes when completing the CST. The CST used in the current study might have been improved by incorporation of additional objective assessments, such as glucose curve parameters, as was done by other investigators [6]. Incorporation of continuous glucose monitoring (CGM) into the study protocol might also have provided an objective method for directly assessing the stability of glucose control over time and for identifying any temporal changes in glycemic control over the intervals covered by glycated protein testing. Since it provides a direct measure of glycemia over time, which is correlated to glycated protein test results, CGM profiles may prove to be superior to clinical assessment methods as an adequate gold standard against which fructosamine and HbA1c results can be evaluated. However, like glycated protein measurements, single glucose curve determinations and CGM profiles provide information about glycemia over a narrow time period and limitations associated with both methods can confound the broader clinical assessment. Thus, discordance between results of individual monitoring tests and the clinical assessment are not unexpected, a fact that underlies recommendations to consider results of multiple monitoring tests in assessing a patient’s clinical diabetes control.

Although HbA1c and fructosamine concentrations are influenced by blood glucose and are expected to increase during prolonged hyperglycemia, these tests display only a moderate positive correlation in diabetic dogs as shown in the current study, similar to earlier findings by others [6, 8]. Since these tests measure blood glycated proteins with different half-lives, different time frames for hyperglycemia is one factor that could impact the correlation between results. Individually, fructosamine and HbA1c are reported to have a significant but relatively poor correlation with a clinical score system that incorporated data from a glucose curve performed at the time of testing [6].

Test correlation with glucose parameters at the time of testing were not determined by the current study. While a positive correlation between glycated protein concentrations and blood glucose concentration is intuitive, differences between the time required for a change in concentration for each measured parameter must be considered when evaluating correlations between results. Correlation between glucose concentration and HbA1c or fructosamine might be expected to be highest when glycemic variability is low or in experimental models where proteins targeted for glycation are exposed to a constant glucose concentration [8, 9]. Likewise, when there is high glycemic variability or glucose concentration fluctuates, correlation with HbA1c or fructosamine might be expected to be low when glucose concentration is determined using random or single sampling or parameters are derived from a single glucose curve. Acute or temporary glycemic perturbation (a stress effect, for example) has been suggested as one cause for discordance between measured glucose and HbA1c concentrations in some dogs [8] and may explain some of the discordant results observed in the current study. Thyroid function was not assessed as part of the current study. Future investigations should aim to evaluate whether fructosamine and HbA1c concentrations, which are time-averaged markers for blood glucose, show improved correlation with similarly time-averaged measures, such as glucose parameters derived from CGM.

Several factors must be considered when interpreting the results of the current study. For the fructosamine test, the cut-off values used to categorize level of diabetes control were those provided by the commercial laboratory that performed the test. Cut-offs for expected HbA1c values and their association with the level of glycemic control in insulin-treated diabetic dogs are not known. The commercial laboratory that performed the HbA1c assay provided a range of cut-offs to assess the likelihood that a dog has diabetes [18]. These guidelines indicate that HbA1c ≤ 4% is expected for normal (non-diabetic) dogs, so this value was selected as a cut-off point for GOOD diabetic control in the current study. In retrospect, this cut-off values is probably too low since few, if any, diabetic dogs would achieve this level of control. A consequence of a stringent cut-off to classify dogs as having GOOD control is that fewer dogs are likely to be correctly classified by the test as having good control, which would tend to reduce agreements between HbA1c and the CST assessment. Indeed, although 14 of 28 dogs were classified as having GOOD control using the CST, only 4 of 28 dogs had HbA1c ≤4%. Among dogs with HbA1c ≤ 4%, two were classified as having GOOD control by clinical score, but HbA1c results were discordant for the remaining dogs, both of which were clinically assessed to have poor control. A value of 8.5% was used as the cut-off for FAIR control. This HbA1c value approximates a mean glucose concentration of about 280 mg/dl [11], a reasonable target for glycemic control in dogs since mean glucose maintained at this concentration would be associated with minimal clinical signs [1]. Values >8.5% were considered consistent with POOR control because these dogs would be expected to have clinical signs as a result of persistent hyperglycemia in a range that exceeds the renal threshold for glucose [1]. The cut-offs selected for the study could have negatively impacted assessment of HbA1c test performance. Cut-offs used to classify study dogs were chosen based on manufacturer guidelines and non-validated clinical targets for glucose rather than from analysis of a ROC. Of 14 dogs classified as having GOOD control by the CS, only 1 had HbA1c <4% but 8 had HbA1c ≤6%, which is the upper limit of the ‘transitional’ range (i.e. a suspicion for mild diabetes) designated by the test manufacturer. An HbA1c value of 6% approximates an estimated glucose concentration of 134 mg/dl [11]. Indeed, when dogs with either GOOD or FAIR glycemic control were combined (effectively increasing the cut-off for ACCEPTABLE control to HbA1c ≤8.3%) test performance was improved. In light of results of the current study, it is appropriate to expect HbA1c values ≤ 8.5% from insulin-treated dogs with acceptable glycemic control determined using a clinical assessment. Previously a cut-off for HbA1c of 6.2% was found to reliably distinguish dogs with naturally-occurring diabetes from non-diabetic dogs [8], suggesting that further stratification of the current results is possible. Values for HbA1c ≤6% should be interpreted as indicating good glycemic control (mean glucose is likely < 150 mg/dl) and values between 6 and 8.5% interpreted as indicating fair glycemic control (mean glucose is likely 150–200 mg/dl). Both serum fructosamine and blood HbA1c results can be affected by factors other than glucose. The fructosamine level is influenced by changes in serum proteins, particularly the serum albumin concentration, and can be altered by disorders that affect the serum protein concentration [19–22]. Some dogs in the current study had albumin concentrations below the reference range but the hypoalbuminemia was judged to be mild in the study population of stable diabetic dogs and unlikely to impact the results. A correlation between serum concentrations of albumin and fructosamine has been reported in dogs. It has been suggested that measured fructosamine should be corrected for albumin in the presence of hypoalbuminemia [23]. However, many of these studies were carried out in non-diabetic dogs, so whether the same correction procedure is warranted in diabetic dogs is not clear. One early study found that the corrected and uncorrected fructosamine provided similar diagnostic value for distinguishing between dogs with or without diabetes [23]. Indeed, a recent study showed that fructosamine displayed overall favorable test performance characteristics when examined using a relatively unselected population of diabetic dogs, some of which had severe concurrent disorders [6], suggesting that the assay is robust in a variety of clinical scenarios.

Anemia has been shown to affect HbA1c in humans and is of particular concern when HbA1c is being used for diabetes diagnosis [24]. However, a recent review concluded that mild anemia does not influence the HbA1c level in humans [24]. A study by Baldo et al. of non-anemic diabetic dogs and diabetic dogs with non-regenerative and regenerative anemias showed that the median HbA1c was significantly higher in non-anemic dogs compared with anemic dogs but HbA1c did not correlate with hematocrit in this group. Variable changes in HbA1c associated with moderate to severe iron deficiency anemia are reported in humans with diabetes [24]. Six dogs in our study had anemia, which was not further characterized. Each of these dogs had a mild anemia (mean hematocrit was 34%) compared to anemic diabetic dogs reported by Baldo et al., which had mean hematocrit values of 22.9% and 21.6% for non-regenerative and regenerative anemias, respectively [6]. It was judged that the mild anemias identified in these six dogs was unlikely to have a significant influence on the HbA1c level and these dogs were retained in all analyses. Although it was determined unlikely that the mild disturbances in albumin and hematocrit present in some dogs would impact the study results, the presence of abnormalities that have the potential to impact glycated protein concentrations should be considered when evaluating an individual patient’s test results. Hypothyroidism can affect fructosamine and HbA1c in dogs and humans but thyroid function was not assess in the current study [25, 26].

The presence or absence of clinical signs is frequently incorporated into assessments of glycemic control in canine diabetes studies [6]. The current study employed a clinical assessment (the CST) as the gold standard for evaluating the usefulness of test results for identifying dogs with various levels of diabetes control. The CST used has been evaluated previously and incorporates owner at-home observations from the 4-weeks preceding the survey and select findings from the physical examination [12]. Similar assessment tools with or without the addition of other data (e.g. results of a glucose curve) have been used by others seeking objective clinically-based evaluation of diabetes control in dogs [6]. In our study, only 5 dogs were classified as having poor control using the CST. While this finding was anticipated since dogs were recruited from a outpatient population with stable chronic diabetes, it is possible that the CST overestimated the number of dogs with fair or good control, which would impact the interpretation of glycated protein test results. For example, test performance could be underestimated if owners consenting to participate in the study introduced positive bias by minimizing the severity of clinical signs, especially polydipsia or polyuria, when completing the CST or providing information they perceived to be associated with good control. Reduction in clinical signs is a reasonable goal for diabetes treatment in dogs but absence of clinical signs is an imperfect gold standard. Even when well regulated, blood glucose will vary and acceptable blood glucose targets during insulin treatment are typically above the reference range for nondiabetic dogs, so a dog may experience hyperglycemia at various times during the day. Because significant hyperglycemia may be present without obvious clinical signs, it is not possible to distinguish between different levels of glycemic control when blood glucose remains below the renal threshold for glucosuria. Direct assessment of glycemia using a continuous glucose monitoring system to provide information about the daily glucose curve, mean daily/weekly glucose concentration, and glycemic variability, which was not employed in the present study, may provide an alternative gold standard for evaluation of HbA1c and fructosamine results.

In conclusion, we have documented the range of HbA1c values expected to occur in insulin-treated dogs with chronic diabetes with various levels of glycemia control. The HbA1c value was shown to be more consistent with clinical assessment of diabetes control than serum fructosamine in clinically stable dogs with chronic diabetes. The HbA1c level correctly classified more dogs than fructosamine but misclassifications occurred using both tests, indicating that HbA1c and fructosamine testing is best used as a component of a comprehensive diabetes monitoring program and test results evaluated in light of all available information about glycemic control.

## Supporting information

S1 Dataset Study dog data. (XLSX)

S1 File The Clinical Survey Tool (CST). (PDF)

## Abbreviations

CST: clinical score tool
DM: diabetes mellitus
eAG: estimated average glucose
HbA1c: hemoglobin A1c
ROC: receiver-operating curve
