# Supplementary material for: Relationship between HbA1c, fructosamine and clinical assessment of glycemic control in dogs
Source: PLoS One. 2022 Feb 25;17(2):e0264275. doi: 10.1371/journal.pone.0264275 (PMC8880912; doi:10.1371/journal.pone.0264275)
Supplement: S1 File — (PDF) [file pone.0264275.s002.pdf]

Owner information and Medical Record#

EVALUATION OF DIABETES CONTROL – Make any additional notes on reverse

<u>Patient history (All refer to changes over previous 4 weeks)</u>

Need for insulin adjustment	Yes<br>No
Change in water consumption	Yes-increased water consumption<br>No
Change in urine frequency/amount	Yes<br>No
Change in appetite/food intake	Change in appetite (increased)<br>No change in appetite
Change in activity level	Change in activity level (decreased)<br>No change in activity level
Increase in body weight	Yes<br>No (no change/decrease)
Signs of hypoglycemia noticed	Yes<br>No
Required treatment for hypoglycemia	Yes<br>No
Compliant with recommended insulin dosing	Yes<br>No

<u>Physical examination</u>

Change body weight $\geq$5%	Yes<br>No
Body condition score	Unacceptable (poor body condition)<br>Acceptable (good body condtion)
Hydration status	Dehydrated<br>Normal hydration
